# Efficacy of pre-operative risk stratification on cataract surgeries in India

**DOI:** 10.6026/973206300211038

**Published:** 2025-05-31

**Authors:** Alisha Elizabeth Alex, Sangeetha T., Inchara N.

**Affiliations:** 1 Department of Ophthalmology, Sri Devaraj Urs Medical College, Tamaka, Kolar, Karnataka, India

**Keywords:** Cataract surgery, risk stratification, small incision cataract surgery (SICS), visual outcome

## Abstract

Cataract remains a leading cause of global blindness, highlighting the importance of effective surgical management. Hence, we evaluated 212 cataract surgeries performed by Indian medical residents to assess the utility of preoperative risk stratification in predicting complications and guiding case allocation. We found that high risk scores were significantly associated with increased intraoperative complications and poor postoperative visual outcomes (p < 0.001). Logistic regression identified preoperative BCVA, risk score and resident experience as significant predictors of complications. Thus, risk stratification is a valuable tool in enhancing patient safety and surgical training in teaching hospitals.

## Background:

Age-related cataract remains a leading cause of preventable blindness worldwide, affecting millions annually with profound socioeconomic implications, particularly in low- and middle-income countries [1]. Cataract surgery, specifically small incision cataract surgery (SICS), is the most commonly performed procedures globally, providing a cost-effective solution to restore vision and improve quality of life [2]. Despite advancements in surgical techniques, complications during cataract surgery continue to pose challenges, potentially impacting visual outcomes and patient satisfaction [3]. Successful surgeries not only restore vision but also contribute to reducing risks of falls, fractures and even depression associated with vision impairment [4]. However, intraoperative complications, such as posterior capsular rupture (PCR), vitreous loss and nucleus drop, can compromise surgical outcomes and necessitate additional interventions [5]. Preoperative risk stratification tools like the Najjar-Awwad, Muhtaseb and Buckinghamshire scores have demonstrated a strong correlation between higher risk scores and increased rates of intraoperative complications [5, 6- 7]. They allow training institutions to ensure patient safety while offering valuable hands-on experience for residents. While risk stratification is a well-established practice in ophthalmology, it allows training institutions to ensure patient safety while offering valuable hands-on experience for residents. Therefore, it is of interest to evaluate the efficacy of a risk stratification system in reducing intraoperative complications and improving postoperative outcomes in resident-performed cataract surgeries.

## Materials and Methods:

This prospective study was conducted on 212 non-consecutive cataract cases performed by residents in the department of Ophthalmology at tertiary care centre from January 2021 to January 2023. All patients of either gender above 40 years of age with cataract were included in the study. Cases with incomplete follow-ups and with history of intraocular surgeries or advanced ocular comorbidities significantly affecting visual outcomes were excluded. After obtaining approval from the Institutional Ethics Committee, written informed consent and demographic data, all patients underwent thorough ophthalmic evaluation followed by small incision cataract surgery under peribulbar anaesthesia. Preoperatively, the clinical findings for each case was systematically analysed to calculate the risk scores using a risk stratification system devised by Muhtaseb *et al.* [5]. This system allocates points to each risk factor (Table 1) according to its potential risk for an intraoperative complication. All the points for risk factors that were documented were added to give a final score for each case and stratified cases were allocated to surgeons with varying levels of experience (Table 2). Risk scores, preoperative and postoperative BCVA, intraoperative and postoperative complications and also the level of resident who operated were tabulated in all the 4 groups and correlated with risk stratification. Data is analysed using SPSS version 22 software with p-value of <0.05 was considered statistically significant.

## Results:

The study included 212 patients who underwent small incision cataract surgery, stratified into four risk groups based on preoperative scores. Group 1 (0 points) comprised 13 patients (6.1%), Group 2 (1-2 points) included 89 patients (42.0%), Group 3 (3-5 points) included 93 patients (43.9%) and Group 4 (6 points or more) had 17 patients (8.0%) (Figure 1 see PDF). These distributions highlight the predominance of patients with moderate risk scores (Groups 2 and 3). Intraoperative complications occurred in 24.5% (52/212) of cases, with a significant increase in higher-risk groups (p < 0.001). Group 1 showed a 23.1% complication rate, Group 2 had 24.7%, Group 3 exhibited 16.1% and Group 4 showed the highest rate at 29.4% (Figure 2 see PDF). Among specific complications, posterior capsular rent (PC Rent) was the most frequent, occurring in 6.6% of cases, followed by zonular dialysis (3.8%), vitreous prolapse (1.9%) and nucleus drop (0.94%) (Figure 3 see PDF). Regression analysis identified several predictors of intraoperative complications. Risk scores were strongly associated with complications (p < 0.001), with higher scores significantly increasing the likelihood of adverse events. Preoperative BCVA also emerged as a critical predictor, with poorer BCVA correlating with higher complication rates (p = 0.016). Additionally, postoperative visual acuity (p = 0.001) and resident training level (p = 0.036) were significant factors. Visual acuity on postoperative day 1 (POD1) was inversely correlated with risk scores. Patients in higher-risk groups exhibited poorer visual outcomes, highlighting the predictive utility of the stratification system. Regression analysis showed that both preoperative and postoperative BCVA significantly influenced risk scores (p < 0.05). The level of resident training played a significant role in the rate of complications. Among the surgeries analysed, 37 (17.5%) were performed by PGY2 residents, 148 (69.8%) by PGY3 residents and 22 (10.4%) by senior residents.

## Discussion:

The study you referenced, involving 212 patients undergoing SICS performed by residents, stratified patients into four risk groups based on preoperative scores. This distribution indicates that a significant majority (86%) of patients fell into moderate to high-risk categories (Groups 2-4). The Indian Multicentre Study analysed 6564 eyes by allotting them in each risk group as Group 1 (44.1%), Group 2 (28.6%), Group 3 (23.9%) and Group 4 (3.3%) [8]. Among experienced surgeons, both phacoemulsification and manual SICS demonstrated low intraoperative complication rates (~1%), whereas among trainees, phacoemulsification had significantly higher complication rates (up to 11.2%) compared to manual SICS (as low as 1.46%), indicating superior safety of manual SICS during surgical training [9]. Similarly a retrospective study conducted at Columbia University, assessed 530 cataract surgeries performed by residents. They grouped the cases according to the Risk score 0: 33.2% risk score 1: 33.8% and risk score ≥2: 33% [6]. This Indian Multicentre study demonstrated a clear correlation between higher risk scores and increased intraoperative complications. The complication rates in the groups were 1.6% in Group 1, 5.7% Group 2, 10.7% in Group 3 and 32.2% in Group 4. The Auckland Cataract Study which utilized both the Muhtaseb and Buckinghamshire risk stratification systems found that the intraoperative complication rates increased with higher risk scores in both stratification systems. Postoperative complication rates and poorer visual outcomes were associated with higher Buckinghamshire risk scores. They inferred that after adjusting for case complexity, complication rates did not significantly differ between residents, fellows and attending physicians, underscoring the utility of risk stratification in training environments. Conversely, the Columbia University Study found no significant association between risk scores and intraoperative complications. But they noted a higher risk scores predicted prolonged corneal edema and other postoperative complications. Since the level of resident training did not significantly affect complication rates, they suggested that risk stratification can aid in safe introduction of surgical procedures to less experienced trainees. The Hungarian Study noted an overall complication rate of 5.4% among 3,272 cases. Out of which Residents: 13.7% and Specialists: 4.8%. [10]. Analysis on the outcome of resident-performed cataract surgeries across different training levels found that surgeries performed by residents in their first semester had a 2.3 times higher likelihood of complications compared to those in their third semester, highlighting the impact of surgical experience on complication rates [11]. The present study reported PCR rate of 6.6% that falls within the range observed in other studies, indicating consistency in this complication's occurrence among resident-performed surgeries. This also could be attributed to the decrease in the surgical skill during the post COVID-19 phase emphasising the importance of skill lab training. A study of 1000 consecutive SICS cases reported a PCR rate of 5.1% in resident surgeries [12]. Another study on phacoemulsification surgeries performed by residents found a PCR rate of 7.5% [13]. Next, zonular dialysis was noted in 3.8 % of cases notably higher than the other studies. Zaidi *et al.* observed that incidence of vitreous loss was 1.1% and that of endophthalmitis 0.1%. [12]. While Magyar *et al.* documented an incidence of 1.1% in resident-performed phaco cases [10]. This might also be a reflection of case selection like more complex cases, less experience seen commonly in the residents, or differences in surgical technique [SICS vs Phaco]. Following PCR, vitreous prolapse was observed in 1.9%, lesser than a study on resident-performed phacoemulsification surgeries which reported vitreous loss in 2.8% of cases [14]. The nucleus drop rate of 0.94% in this study is comparatively higher than other studies [15- 16] which could be attributed to risk factors such as mature cataracts, poor red reflex, pseudo-exfoliation and small pupil especially in high-risk stratified cases. The findings of this study demonstrate that preoperative risk stratification plays a critical role in predicting intraoperative complications and optimizing surgical outcomes in small incision cataract surgeries performed by residents. Authors have strongly supported this showing that matching case difficulty with surgical experience reduced intraoperative complications significantly, ensuring patient safety and providing residents with a structured learning pathway [17]. Another study highlighted that a comprehensive cataract surgery complexity score system can guide appropriate case selection to match trainee experience, minimizing complications and optimizing outcomes [18]. Further, higher risk scores, which included factors like poor preoperative BCVA, were predictive of prolonged corneal edema and other postoperative complications [6].

## Conclusion:

The importance of preoperative risk stratification in cataract surgeries by Indian medical residents showing its effectiveness in predicting and reducing intraoperative complications is reported.

## Figures and Tables

**Table 1:** Patient characteristics used in the scoring protocol

**Category A (No Points)**	**Category B (1 Point Each)**	**Category C (3 Points Each)**
No additional risk factors carried by the patient	Corneal scarring, Small pupil (3mm), Shallow anterior chamber (depth 2.5mm) , Age >88 years, High ametropia (>6 D of myopia or hyperopia), Posterior capsule plaque, Posterior polar cataract, Previous vitrectomy, Miscellaneous risks assessed by the surgeon (eg, poor position of eye/patient).	Dense/total/white or brunescent cataract, Pseudoexfoliation, Phacodonesis.

**Table 2:** Allocation of cases categorised into the risk group to the surgeons

**GROUP**	**RISK**	**POINTS**	**SURGEONS LEVEL**
I	No added risk	0	Junior resident (PGY2)
II	Low risk	2-Jan	Junior resident (PGY3)
III	Moderate risk	5-Mar	Senior resident
IV	High risk	>6	Senior surgeon
